# Neuro-otology- some recent clinical advances

**DOI:** 10.1007/s00415-016-8266-1

**Published:** 2016-09-15

**Authors:** Miriam S. Welgampola, Gülden Akdal, G. Michael Halmagyi

**Affiliations:** 1 Neurology Department, Royal Prince Alfred Hospital, Sydney, Australia; 2 Neurology Department, Dokuz Eylül University Hospital, Izmir, Turkey

**Keywords:** Vertigo, Head impulses, vHIT, VEMP

## Abstract

Vestibular disorders manifesting as vertigo, chronic dizziness and imbalance are common problems in neurological practice. Here, we review some recent interesting and important advances in diagnosis of vestibular disorders using the video head impulse test and in the management of benign positional vertigo and migrainous vertigo.

## The history in the patient with vertigo

By ‘dizzy' (synonym ‘giddy')—from late Middle English *gidig* meaning ‘insane’ or ‘possessed by a god’, we mean any complaint related to balance, vestibular, or non-vestibular or both. Other modern English words for balance problems are: vertigo (from Latin *vertere*, to turn), an illusion of rotation, normal, of course, after spinning, and then suddenly stopping; swaying (as when standing, while drunk), staggering (as when walking while drunk) rocking (as when standing up in a moving train carriage), bobbing (an up-and-down linear motion), and dropping. Two caveats: (1) To take a balance history via an interpreter, even from an educated, motivated patient, can be both frustrating and misleading. (2) To avoid making, what for an otologist would be an elementary mistake, neurologists need to order and be able to interpret audiograms.

## The patient with recurrent acute vertigo attacks

In patients seen electively by appointment, complaining of what after careful interrogation sounds like recurrent acute vertigo attacks, the differential diagnosis is basically limited to benign positional vertigo (BPV), Meniere’s disease (MD) or vestibular migraine (VM) [1]. Patients who start to have vertebrobasilar transient ischaemic attacks predominantly manifesting with vertigo will usually have a stroke long before their appointment comes around [2].

## Benign positional vertigo (BPV)

BPV is the commonest cause of recurrent vertigo. The vertigo attacks are brief, usually lasting seconds, rarely minutes, triggered by bending down, looking up or rolling over in bed [3]. The elderly might present with falls getting out of bed [4]. BPV is caused by otoconia dislodged from an otolith macula, moving within a semicircular canal duct (“canalithiasis”) [5] or becoming attached to its cupula (“cupulolithiasis”). As the head changes position with respect to gravity, movement of these ectopic otoconia, within the canal duct or on the cupula, under the influence of gravity, activates or inhibits canal afferents, producing vertigo and nystagmus with an axis that is orthogonal to the affected canal plane [6].

## Posterior canal BPV

**Fig. 1:**
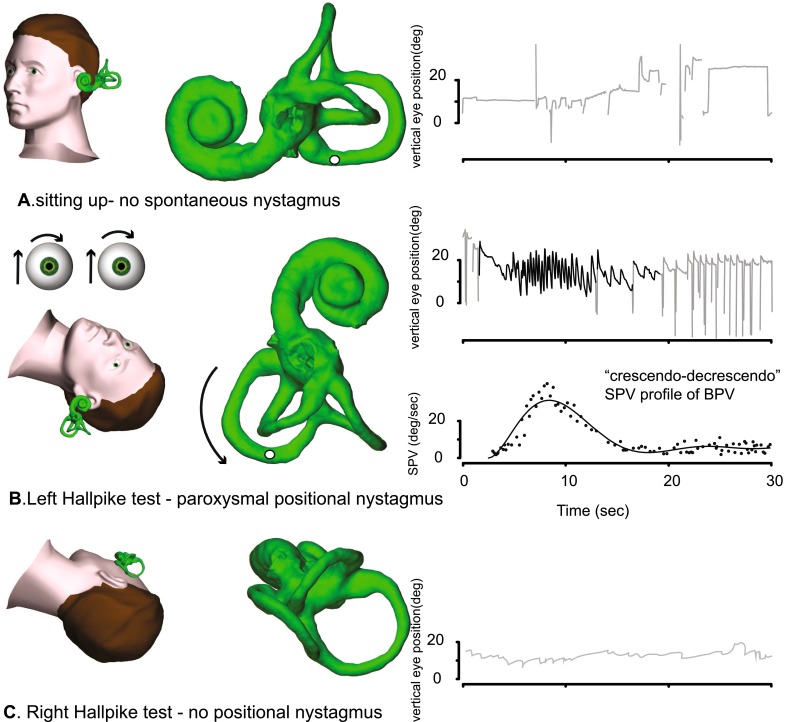
The typical nystagmus profile of right posterior canal BPV. When the subject is upright (**a**), no nystagmus is seen. In the right Hallpike position (**b**), after a latency of 2–3 s, a paroxysm of upbeating torsional geotropic nystagmus is seen, with a crescendo–decrescendo vertical slow-phase velocity (SPV) profile

Posterior semicircular canal (PC) BPV accounts for up to 90 % of all BPV presentations. In the common variety, there is geotropic, torsional-upbeating nystagmus in the Dix–Hallpike test, indicating that the otoconia are falling downwards in the excitatory direction, away from the posterior canal cupula of the lowermost ear (Fig. 1). Diagnostic criteria for posterior canal BPV are [7]: (1) recurrent attacks of positional vertigo or dizziness provoked by lying down or turning over while supine; (2) duration less than 1 min; (3) positional nystagmus elicited after a latency of a few seconds by the Dix–Hallpike test or by the side-lying test; (4) torsional-upbeating (posterior canal plane) nystagmus lasting less than 1 min; and (5) no other disorder that accounts for these findings [3]. Investigations are indicated only when an underlying cause for BPV is suspected [8].

Standard PC BPV is treated by either the Epley [9] or the Semont [10] maneuver, not just by doctors but also by physiotherapists [11] and audiologists [12]. Some patients learn to treat themselves. A single Epley maneuver has a success rate of up to 80 % [13, 14]; the success rate increases to over 90 % with four repetitions on the same day. Previous head trauma and prolonged bedrest are risk factors for a poor outcome after a single Epley maneuver, while BPV secondary to some inner ear disease is not [15]. After a simple maneuver, in which the subject first lies supine for 3 min, then on the unaffected shoulder for some hours, the success rate is 89 % after 1 week and 100 % after 2 weeks [16]. Despite unequivocal evidence, over 20 years, of the efficacy of such simple treatment for such a common disorder and two independent practice guidelines published 8 years ago [17, 18], as recently as 4 years ago, only 4 % of patients presenting to emergency with what turned out to be BPV even had a Dix–Hallpike test; most of the other 96 % were investigated with blood tests and brain CT and prescribed antiemetic tablets [19]!

## Apogeotropic posterior canal BPV

Rarely, a BPV patient has torsional downbeating, rather than the usual upbeating, nystagmus in the Dix–Hallpike position, is taken to have anterior semicircular canal (AC) BPV, but soon develops nystagmus typical of PC BPV of the other side [20]. These patients are thought to have otoconia in the distal, non-ampullary arm of the posterior canal, close to the common crus. Dix–Hallpike testing is assumed to have produced movement of the otoconia towards the ampulla, inhibiting PC afferents and producing an inhibitory, i.e., torsional-downbeating, nystagmus. This positional nystagmus can be provoked in either right or left Dix–Hallpike positions, or in the head-hanging position, and sometimes, even in a side-lying position. There is no latency, but a crescendo–decrescendo time-course, and the nystagmus is not completely exhaustible. Rising to the upright position does not reverse the nystagmus direction, and it does not fatigue on repeated positional testing. These results make sense, since excitation of the AC on one side will produce the same nystagmus as inhibition of the PC on the other. Two treatments have been proposed: the second half of the Semont maneuver, which the patient begins by sitting upright with legs hanging over the edge of the bed, the head rotated toward the unaffected ear; then whilst maintaining this head position, lying onto the unaffected side, allowing the otoconia to fall into the common crus and finally into the vestibule [21]. The second treatment, termed the “45-degree forced prolonged position,” requires patients to lie on the unaffected side with the head turned 45° downwards, to bring the non-ampullary arm of the affected posterior canal into a draining position, and to maintain this position for 8 h. This treatment worked in 68 % of 16 patients.

## Horizontal canal BPV

**Fig. 2:**
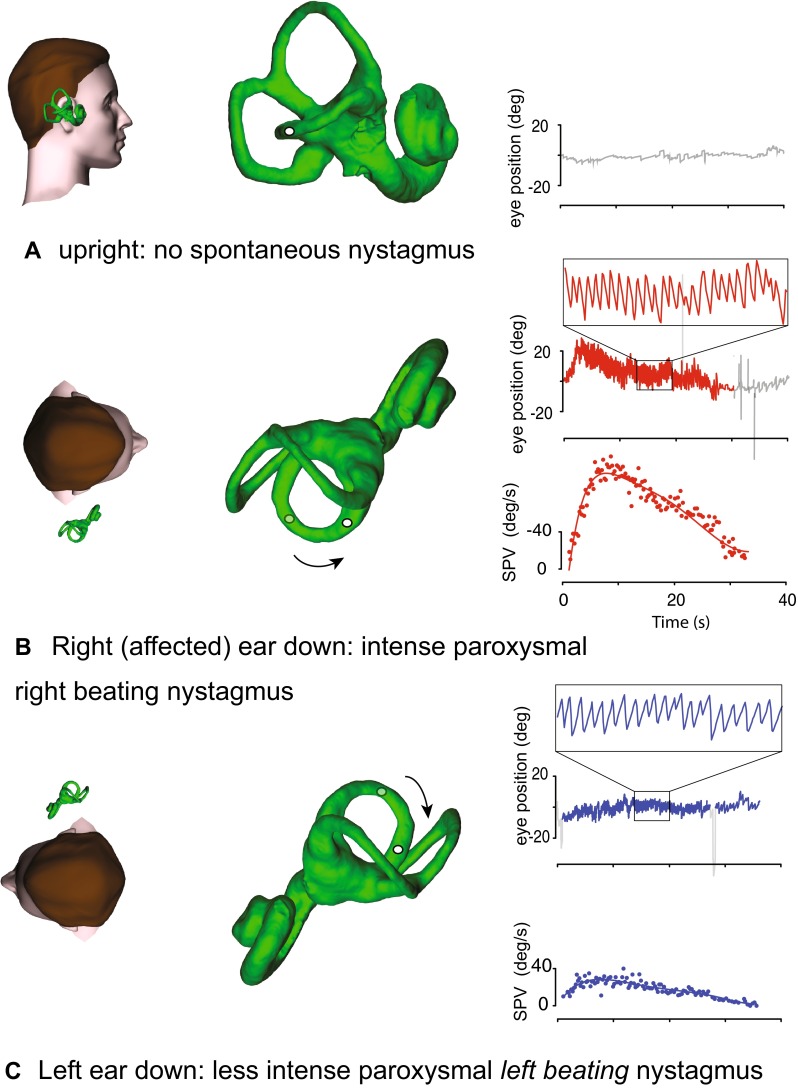
Right horizontal canal BPV. When the subject is upright (**a**), no nystagmus is seen. In the right “side-lying” position (**b**), after a latency of ~1 s or less, a paroxysm of horizontal geotropic nystagmus is seen, with a crescendo–decrescendo slow-phase velocity profile. A similar but less intense paroxysm of horizontal geotropic nystagmus is seen with the unaffected left ear down

Horizontal semicircular canal (HC) BPV accounts for about 10 % of all BPV presentations (Fig. 2). HC canalithiasis produces a paroxysmal positional nystagmus beating toward the lowermost ear (geotropic nystagmus). Nystagmus slow phase velocity is higher when the affected ear is lowermost, and it has shorter onset latency than PC BPV, a crescendo–decrescendo pattern and a relatively longer duration, still less than 1 min [6, 22]. HC cupulolithiasis is characterized by persistent apogeotropic positional nystagmus, maximal with the unaffected ear lowermost [22]. Persistent, geotropic direction-changing positional nystagmus, without latency, could originate from a “light cupula,” i.e., a cupula with a lower than normal specific gravity [23–25]—similar to that in phase 1 of alcohol-induced positional nystagmus. About 40 % of HC BPV patients have migraine with geotropic or apogeotropic nystagmus [22, 26–28]. Those of our colleagues who do not own an examination couch, can examine these patients quite well in a chair using the “bow-and-lean” test [29].

The immediate (1 h) and long-term (1 month) efficacy of various repositioning maneuvers for HC BPV were recently compared in a randomized, prospective, and sham-controlled study [30]. The immediate efficacy was comparable for barbecue (69 %) and Gufoni (61 %) manoeuvres, and significantly better than a sham treatment (35 %). The cumulative therapeutic effects were also significantly better for both manoeuvres than for the sham maneuver, thus providing class I evidence for the efficacy of both treatments for horizontal canalithiasis.

## BPV after acute vestibular syndrome

When BPV accompanies a recent acute vestibular syndrome, its etiology should be confirmed with video head impulse testing (vHIT) for semicircular canal function, vestibular evoked myogenic potentials (VEMPs) for otolith function, and with audiometry. With BPV after vestibular neuritis, there can be impaired ocular VEMPs (oVEMPs) from the utricle and horizontal plus anterior SCCs vHITs but normal cervical VEMPs (cVEMPs) from the saccule [31]. In contrast, with BPV after labyrinthitis or labyrinthine infarct, there is sudden hearing loss, and there can be prolonged geotropic or apogeotropic horizontal positional nystagmus (as in cupulolithiasis) refractory to treatment, and also abnormal posterior SCC vHIT [32, 33]. Apogeotropic horizontal nystagmus could be due to post-labyrinthitis inflammation within the ampulla [34] and geotropic nystagmus to a light, “floating” cupula [25].

## Positional vertigo without positional nystagmus

If the story sounds like BPV, but there is neither positional vertigo, nor positional nystagmus—with a correctly done Dix–Hallpike test—it is best to see the patient again [35] rather than to order tests. An unequivocal diagnosis of BPV requires positional nystagmus. However, some patients who have no nystagmus during the Dix–Hallpike test, still complain of brief paroxysms of vertigo after coming up, and have retropulsion and measurable oscillation of the trunk at the same time, possibly due to otoconia in the short arm, i.e., on the utricular side, of the posterior SCC. Some of these patients can be treated effectively with repeated sit-ups from the Dix–Hallpike position [36].

## Positional nystagmus without positional vertigo

BPV can be overdiagnosed if its unique attribute, canal-plane nystagmus, is not sought. With removal of visual fixation, an asymptomatic low-amplitude positional nystagmus is common in healthy subjects [37]: about 55 % have upbeating nystagmus in the Dix–Hallpike position, with slow-phase velocity up to 5°/s. Unilateral horizontal positional nystagmus is also common (23 % geotropic, 32 % apogeotropic), although bilateral, direction-changing apogeotropic or geotropic nystagmus is rare (less than 5 %).

## Central positional vertigo and nystagmus

Paroxysmal positional vertigo and nystagmus can occur in posterior fossa lesions [38] and can resemble AC BPV [36]. Downbeating nystagmus upon supine head-hanging, upbeating nystagmus upon returning from supine to upright position, and apogeotropic horizontal–torsional nystagmus during the Dix–Hallpike or the supine head-roll test, all occur [39], mainly with cerebellar strokes and tumors involving the nodulus and uvula. The direction of central paroxysmal positional nystagmus, unlike the direction of peripheral, i.e., benign, paroxysmal positional nystagmus, aligns with the vector sum of the rotational axes of the semicircular canals that were being inhibited during each positioning: thus, for example, straight head hanging would inhibit both anterior canals: the nystagmus is directly upbeat with no latency, a rapid crescendo phase which decreases exponentially with a time constant of 3–8 s, a normal value for nystagmus arising from the vertical canals.

## Mechanical rotators for treating BPV patients

There can be practical problems diagnosing and treating even a simple case of unilateral PC BPV if, for example, the patient is 80 years old, weighs 120 kg and has Parkinson’s disease. It is then impossible to do a proper Epley (or Semont) maneuver, or even an accurate Dix–Hallpike test, on a narrow examination couch, jammed in the office corner, against the wall. There are two solutions to this problem. (1) A home visit. Testing and treating such patients in their own home, on their own double bed. With video Frenzel glasses, it is possible not only to check the positional nystagmus and so monitor the treatment, but also to show any skeptical family member that there really is something wrong. (2) A multiple axis patient rotator. The Epley Omniax Rotator and the TRV chair are expensive mechanical repositioning devices that allow for effective treatment of BPV that is refractory to bedside treatment or involves multiple SCCs, especially in those with the physical limitations described above. Both devices hold promise but have not yet been compared against bedside maneuvers in randomized trials [40].

## Meniere’s disease

**Fig. 3:**
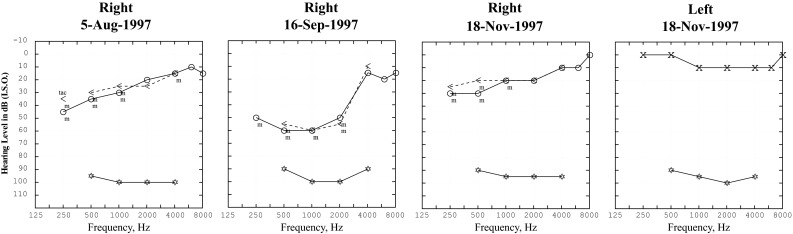
Three sequential pure-tone audiograms from the right ear of a 19-year-old female with vertigo attacks due to Meniere’s disease, showing the typical fluctuating, low-frequency, sensorineural hearing loss. First audiogram is 1 month before a vertigo attack, second audiogram is 1 day after a vertigo attack and the third is 2 months after the attack. Compare with the normal audiogram from the unaffected left ear. The acoustic reflex thresholds—shown with star (*) symbols at the bottom of each graph, do not change with the increase in subjective pure-tone threshold (30 dB at 1 kHz) on 16-Sept-1997, indicating recruitment, characteristic of a cochlear hearing loss. Masked (m) bone conduction thresholds are shown with (<) symbols; there is no conductive component the hearing loss

So, if it is not BPV, then is it MD or is it VM? In a young, otherwise, well patient, the diagnosis of MD is usually easy—there is a unilateral tinnitus and fullness with a fluctuating, low-frequency, cochlear-type sensorineural hearing loss (i.e., intact acoustic reflexes) which might not be obvious during, or even after, the first few vertigo attacks, but will be eventually [41]. During the first few attacks, the patient is usually too dizzy to notice the hearing problem, and certainly cannot cooperate with an accurate audiogram. There is no other cause of a low-frequency hearing loss that keeps getting better (Fig. 3). Accurate audiological evaluation and interpretation, in cooperation with an experienced otologist, is essential to make the diagnosis. Difficulties arise when the patient has a pre-existing, unrelated hearing loss: low-frequency conductive (middle ear effusion), mid-frequency sensorineural (congenital) or high-frequency sensorineural (age/noise induced), or if the patient has bilateral MD. Drop attacks—the patient just drops to the ground—occur in MD and also in some non-MD aural diseases, but not in migraine [42]. Neurologists rarely remember to order audiogram and vestibular function tests, as well as EEG and ECG, in patients with drop attacks [43]. Repeated room tilt illusion attacks—suddenly the whole visual world is tilted or even inverted—might be a related phenomenon: these occur with both MD and migraine, and possibly even with vertebrobasilar TIAs [44, 45].

In between MD attacks, there can be unilateral vestibular impairment, from the deaf ear, of VEMPs [46], of caloric responses, but interestingly not of the head impulse test [47], perhaps because endolympatic hydrops abolishes convective fluid movement [48]—more about this below.

During acute MD attacks, there will be a wild nystagmus, at first beating toward the affected side (excitatory nystagmus), then toward the normal side (paretic nystagmus) and then again toward the affected side (recovery nystagmus), all enhanced by head-shaking (in a brave patient) [49]. Both the VEMP and the vHIT can be overactive [50] or the vHIT can be impaired [51]. Settings of the subjective visual horizontal (or vertical) will deviate, usually in the same direction as the nystagmus slow phases [52]. However, it is exceptional to see a patient during an entire attack, and even if one does, without knowing from the hearing loss which is the affected ear, the vestibular signs do not accurately lateralize the MD.

The vertigo attacks in MD can usually be stopped [53]. Therapeutic unilateral vestibular deafferentation of the affected ear with vestibular nerve section, surgical labyrinthectomy or intratympanic gentamicin injections can do this but at the risk (~25 %) of producing mild, but permanent, subjective imbalance [54], annoying for both patient and doctor, and needing immediate intensive vestibular rehabilitation [55]. Intratympamic dexamethasone might be just as good and should not produce imbalance [56]. A low-sodium diet is traditional [57], endolymphatic sac surgery is controversial [58], drugs such betahistine [59], cinnarizine and dimenhydrinate [60] hopeful.

## Vestibular migraine

Many patients with migraine headaches also have balance problems, including vertigo attacks [61–65], and many patients with vertigo attacks, or other balance problems, also have migraine headaches [66]. There are now official criteria for the diagnosis of VM [64, 67], even though many migraineurs have other, unofficial, balance problems, such as chronic subjective dizziness [68], motion sensitivity [69], motion sickness [70], constant rocking sensations (mal-de-debarquement) [71], room-tilt illusion [45], and generalized imbalance [72]. And, perhaps as a consequence of the vertigo attacks, VM patients have psychological problems such as anxiety [73], panic attacks [74], and phobias [75]. Children commonly have VM [76] but rarely have BPV [77].

**Fig. 4:**
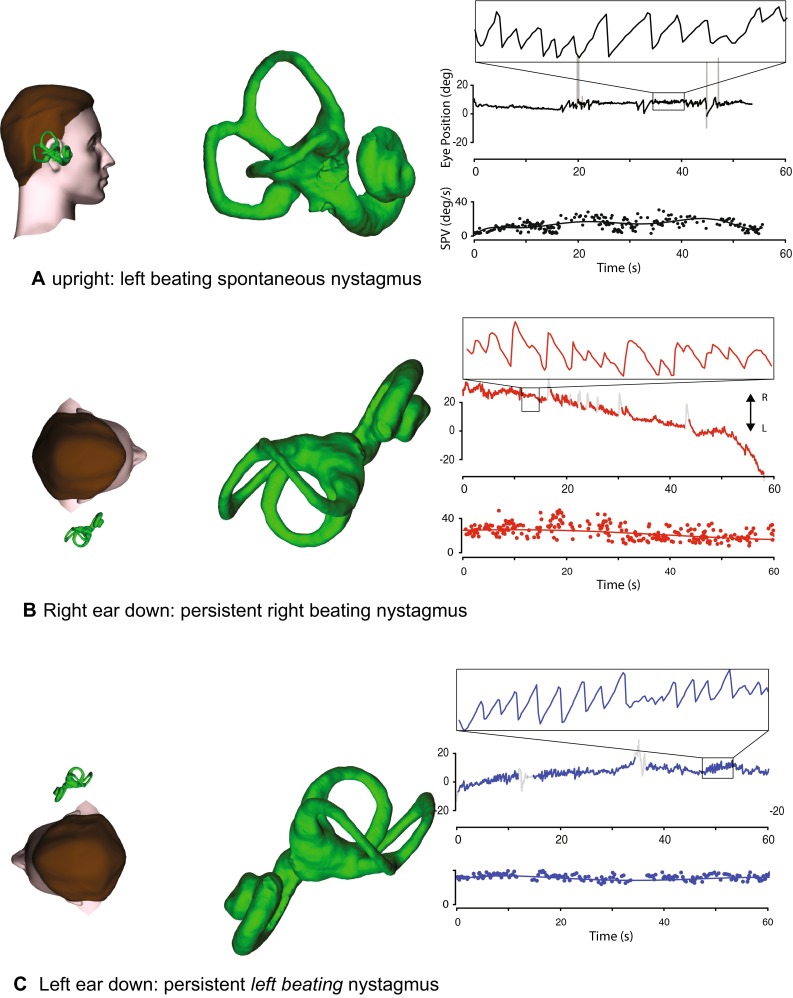
Atypical positional nystagmus in a subject with clinically definite vestibular migraine. Sitting upright, left-beating horizontal spontaneous nystagmus is seen. With either ear down, persistent horizontal geotropic nystagmus, which has a “flat” SPV profile, is seen

The neurological and vestibular examination should be normal in between VM attacks. During an attack, there might be spontaneous nystagmus, positional nystagmus (Fig. 4), direction-changing or direction-fixed [78] that can sometimes be hard to distinguish from BPV, MD or from a central vestibulopathy [79]. When patients have both MD and migraine things really get complicated [80–82].

Also, patients can have headache with their BPV [83] and those who have migraine are more likely to have BPV than those who do not [84]; those who have idiopathic BPV are more likely also to have migraine than those who have post-traumatic BPV [85].

Although there is no solid evidence that any treatment is of benefit in VM [86], patients are usually treated, with drugs used for treatment and prevention of migraine headaches: betablockers, pizotifen, tricyclics, anticonvulsants (topiramate, lamotrigine, and valproate); cinnarazine, flunarazine, and triptans [86].

## Video head impulse testing

A short, fast, head acceleration tests semicircular canal afferents and the brainstem in much the same way as patellar tap tests 1a afferents and the lumbar cord; head impulses test the vestibulo-ocular reflex (VOR) in response to rapid head accelerations. These responses are hard-wired into the neurophysiology of the semicircular canals and the brainstem; they depend on the resting rate and on–off asymmetry of primary canal afferents and their robust di- or tri-synaptic projections via the vestibular nuclei to the oculomotor nuclei. The head impulse test (HIT) can detect severe loss of function of any single SCC [87, 88].

It is sometimes, but not always, possible to detect in the clinical HIT, the characteristic compensatory “catch-up” saccades that result from a defective VOR. This test depends, as does any other aspect of the neurological examination, both on clinician skills and patient co-operation. If the catch-up saccades have a short latency and so occur, while the head is still moving rather than just after it has stopped moving, they will be “covert,” that is, invisible to the clinician [89]. Until recently, objective measurement of the defective VOR in the HIT has been possible only with a complex method limited to research laboratories: scleral search coils [90]. There are now several commercially available, video-based systems (most head-mounted, one wall-mounted) [91–93], with which a clinician can measure the VOR from each of the six canals in a reasonably co-operative adult or child [94, 95] in about 10 min. Audiologists [96] and physiotherapists [97] are already doing so. Here, we consider four common clinical situations in which the video head impulse test (vHIT) can help with diagnosis.

**Fig. 5:**
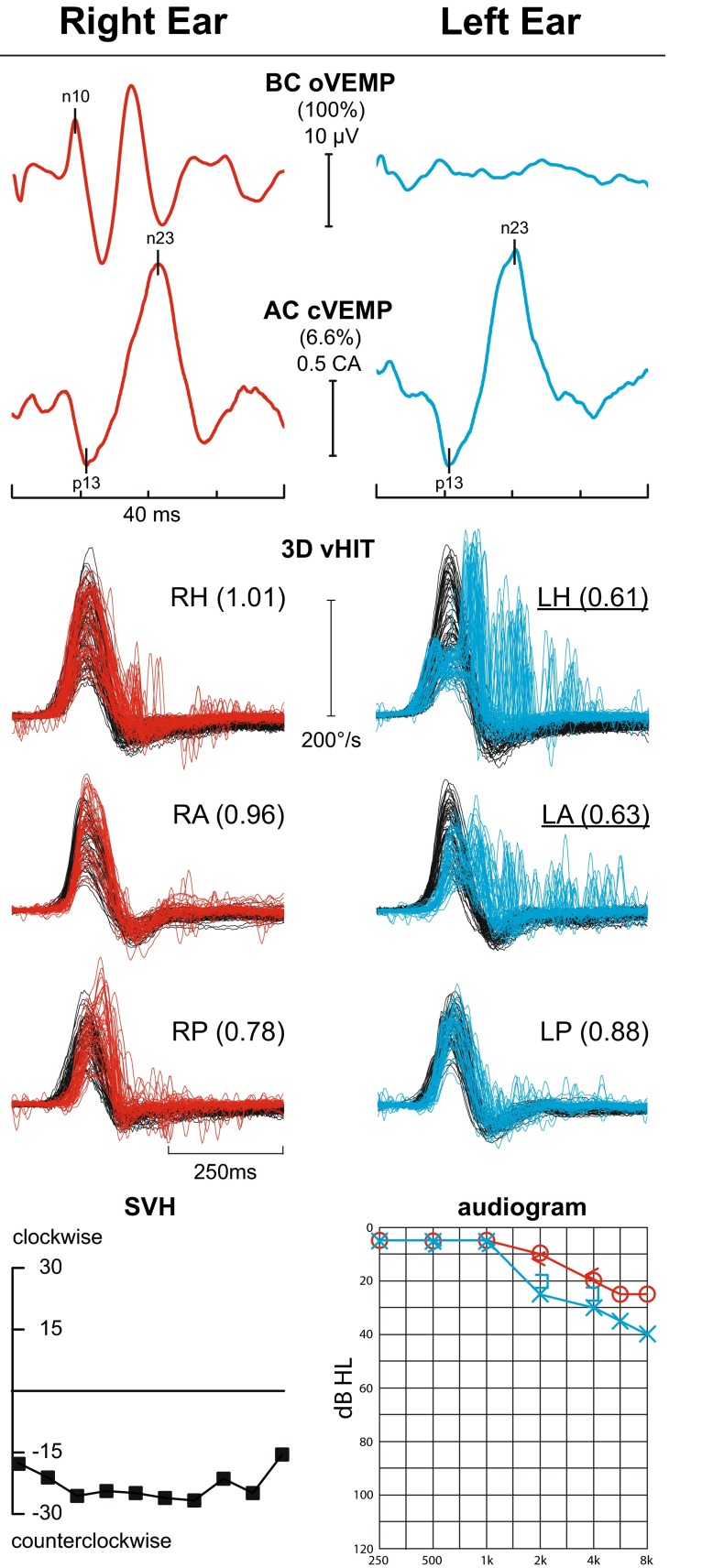
Vestibular Neuritis. Vestibular test profile of a patient with left vestibular neuritis who presented with isolated acute spontaneous vertigo lasting 3 days. The vHIT shows reduced gain from the left horizontal (0.61) and anterior (0.63) semicircular canals with abnormal catch-up saccades. The ocular VEMP, indicating dynamic utricular function, is absent from the left ear but cervical VEMPs, indicating dynamic saccular function, are symmetrical. cVEMP amplitudes divided by background rectified EMG activation (corrected amplitude "CA") show only a 6.6 % asymmetry—abnormal in our laboratory is >35 %. The subjective visual horizontal which tests the left-right balance of static utricular function show a very large (28 deg) counterclockwise (i.e., towards the left ear) offset indicating reduction in left utricular function. The audiogram shows only a mild, slightly asymmetrical (left > right) high frequency hearing loss, almost certainly entirely unrelated to the vestibular neuritis

**Fig. 6:**
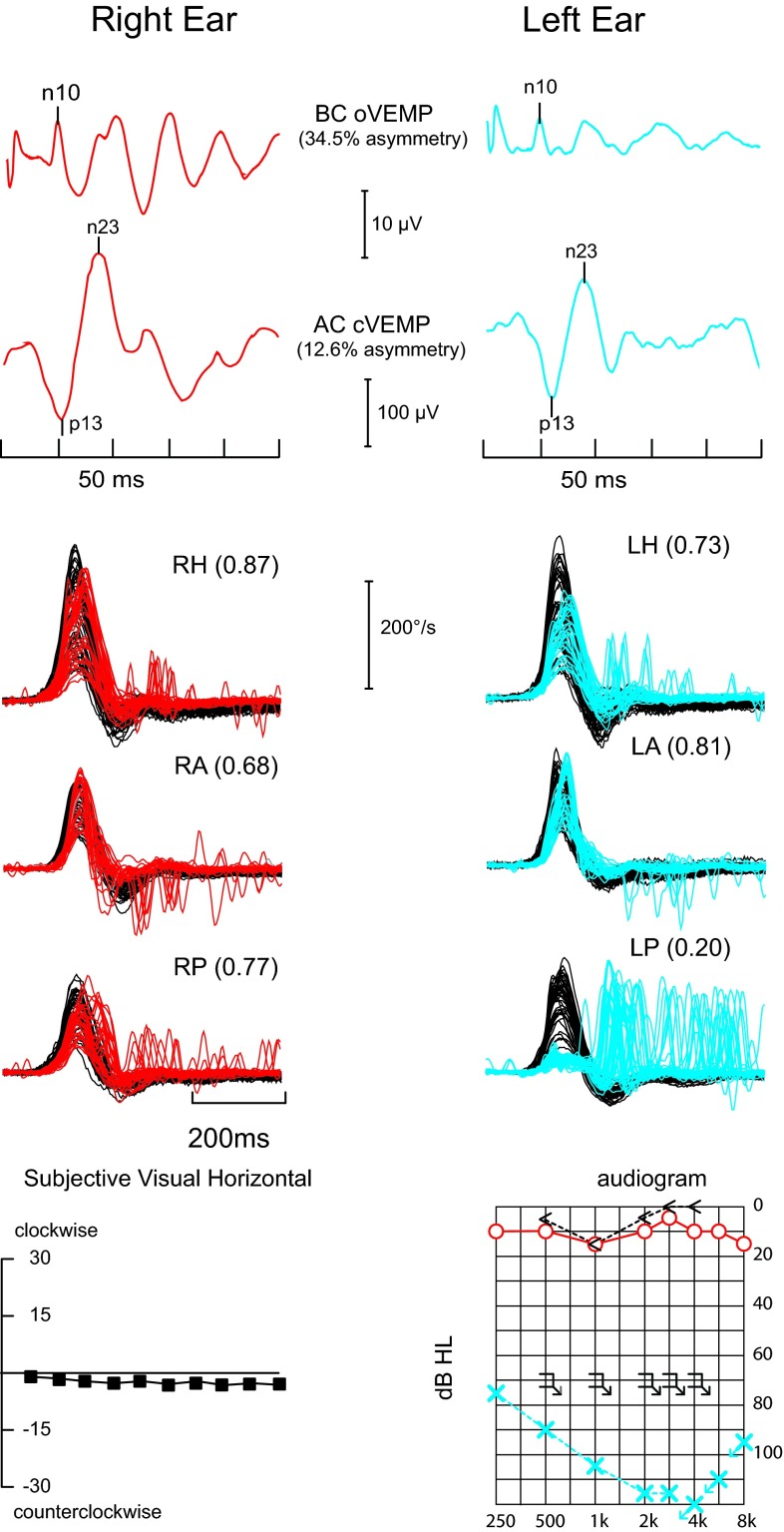
The vestibular test profile of a patient with left labyrinthitis/labyrinthine infarction presenting with sudden severe left hearing loss—symbolized with blue crosses; (]) symbols indicate no response to bone-conduction testing at 70 dB and acute spontaneous vertigo lasting days. The left posterior canal function is selectively impaired. Although both the cervical and ocular VEMPs appear smaller from the affected ear, they fall within the normal range

(i) *vHIT during an acute vestibular syndrome* Patient is seen, usually in the Emergency Room, during her first-ever attack of acute, spontaneous, isolated vertigo. Assuming there is no simultaneous acute unilateral hearing loss (neurologists rarely ask and almost never test for hearing loss), the two main diagnoses are vestibular neuritis and cerebellar infarction. A competent, focused clinical examination such as HINTS can often distinguish between the two [98, 99]. It is not possible to diagnose acute vestibular neuritis without showing acute unilateral loss of function in one, two, or all three SCCs. Loss of only HC and AC function suggests involvement of only the superior vestibular nerve; this can be corroborated by finding loss of ipsilateral oVEMPs with preservation of cVEMPs (Fig. 5). [31, 100–109]. Patients with superior vestibular neuritis will have contraversive 3rd degree horizontal–torsional spontaneous nystagmus and often, but not always, unilateral impairment of the clinical HIT. This is where vHIT is useful: objective, quantitative measurement of the VOR from all six SCCs, showing and documenting that there really is unilateral impairment of SCC function (Fig. 6).

Inferior vestibular neuritis [87], affecting just the PC—corroborated by finding absent cVEMPs (from the saccule) and preserved oVEMPS (from the utricle) [103, 104]—can only be confidently diagnosed with vHIT. In contrast to acute vestibular neuritis, a cerebellar infarct rarely impairs the VOR, so the patient will have a normal or near-normal vHIT [105, 107, 110]. This logic is counter-intuitive: it is the normal test, in this case the normal vHIT, that indicates a potentially serious condition—cerebellar infarction, with a 20 % chance of foramen magnum herniation needing immediate posterior fossa decompression to prevent death [111], and the abnormal test, the vHIT, that indicates a safe-to-discharge condition—vestibular neuritis. Two other conditions that can produce acute, isolated, spontaneous vertigo—MD and migraine—also do not show impairment of the VOR on vHIT, and they can be hard to differentiate from cerebellar infarction when seen in the acute phase. However, it is exceptional for there not to be unilateral tinnitus, fullness and low-frequency deafness in MD, even during the first attack—see above. On the other hand, most patients with a MD vertigo attack are too busy being dizzy to complain about or even to notice the hearing problem, especially in the masking din of most Emergency Rooms, especially if nobody bothers to ask about it and if nobody is able to test for it. A severe, first-ever, migrainous vertigo attack might be more difficult to distinguish from cerebellar infarction—even by an experienced neuro-otologist. A negative DW MRI [112] and a detailed headache history, once the patient has recovered, are probably the only way. The editor of Practical Neurology has given us a clear and concise personal account of what it is like to have, and to have had, acute vestibular neuritis [113].

(ii) *vHIT after an acute vestibular syndrome* The patient is seen days, weeks—whenever she can get an appointment—after such an attack. She is now asymptomatic, simply wanting to know what happened. Or perhaps complaining of persisting imbalance, because she had acute vestibular neuritis and while her brainstem has compensated [114], she has not recovered peripheral vestibular function, and now has chronic vestibular insufficiency [115] comprising a feeling of imbalance, a positive foam Romberg test and head movement oscillopsia. Or because she actually had a cerebellar infarct. Alternatively, she could be complaining of further but less severe, vertigo attacks; if the attacks are spontaneous, it might be that she actually has MD; if the attacks are positional, it might be PC BPV as a result of the vestibular neuritis [8, 116]. After acute vestibular neuritis, only some patients recover canal function—as judged by vHIT or by caloric test or rotational testing [117, 118]. If the vHIT is still impaired on one side, the diagnosis of vestibular neuritis can be safely made in retrospect, but some patients do recover vestibular function—it probably has nothing to do with steroid treatment [119]—and have a normal vHIT (and caloric), so that the distinction between recovered (as opposed to simply compensated) vestibular neuritis [118, 120] and cerebellar infarction cannot now be made clinically, and will need MRI. If that too is normal, there is a diagnostic problem. Is this an MR negative cerebellar infarct [112], a cerebellar TIA (is there such a thing?) or a recovered vestibular neuritis? Could the patient have had paroxysmal AF with a cerebellar embolus [121, 122]? How far to go? There are no easy answers.

(iii) *vHIT with recurrent vertigo attacks* The patient is seen well, but complaining of recurrent vertigo attacks, either spontaneous or positional. If the attacks really are vertigo, then VM, MD, and BPV are just about the only possible diagnoses. Rarely, recurrent vertigo is the presenting symptom of Stokes–Adams attacks [123]. Unfortunately, most patients who have started to have isolated vertigo attacks from vertebrobasilar TIAs will stroke out long before their appointment comes around [2]. Recurrent vertigo attacks are the most common vestibular cases in office practice, but a vHIT rarely helps as it is usually normal, even in Meniere’s disease [47, 48, 124]. Nonetheless, it is still worth doing: for example, occasionally BPV is secondary to some inner ear disease [8], and the vHIT will be abnormal.

(iv) *vHIT in chronic imbalance* There are many possible causes, especially in the elderly, of chronic imbalance: neurological (sensory neuropathy, extrapyramidal disorders, orthostatic tremor, normal pressure hydrocephalus—to name just a few), psychological [125, 126], musculoskeletal and what concerns us most here—vestibular. Chronic vestibular insufficiency can be due to severe unilateral [127, 128] or moderate, symmetrical or asymmetrical, bilateral vestibular impairment [129, 130]. The patient with chronic vestibular insufficiency has no symptoms while sitting or lying but feels off-balance as soon as she stands and more so when she walks [131]. There will be no clinically detectable impairment of gait or stance—even with eyes closed and feet together—a negative Romberg test, but if the patient tries to stand on a soft surface, say a foam mat, then she will sway and fall if not caught—the positive foam Romberg test—almost diagnostic of vestibular impairment, (patients with proprioceptive impairment already have a positive Romberg test on the firm surface of the floor) [132]. The other symptom the patient will have is vertical oscillopsia during vertical head-shaking (due to impairment of the vertical VOR). The patient might even volunteer, or at least admit, that she has to stop in order to see clearly, and having the examiner shake her head up-and-down will drop her vision by at least three lines on a Snellen chart. In these patients, the vHIT is the most useful vestibular test.

Bilateral vestibular impairment needs to be severe to be detected on caloric or rotational tests, as both tests have such large normal ranges. Although unilateral vestibular impairment, of one lateral SCC, even if only mild, can be detected by caloric testing, if only mild, it will not produce imbalance. In contrast vHIT has a tight age-adjusted normal range [91], and is the test of choice for measuring whether vestibular function is impaired sufficiently to produce imbalance on its own. A common cause of an isolated severe unilateral vestibular loss presenting with chronic vestibular insufficiency is an unrecognized previous attack of acute vestibular neuritis [133]—some patients do not take much notice of vertigo. If there is definitely no history of a previous vertigo attack, then a chronic progressive cause of unilateral vestibular loss such as a vestibular schwannoma—hearing should also be impaired—needs to be excluded [134–136]. The cause of bilateral isolated vestibular loss, if not hereditary [137], not due to bilateral sequential vestibular neuritis [101] or gentamicin toxicity [127, 129] usually remains undiagnosed. If accompanied by hearing loss then many more diagnoses are possible: again hereditary [138], but also acquired, usually sinister diseases, such as superficial siderosis [139] and leptomeningeal carcinomatosis [140]. If there are also proprioceptive and cerebellar impairments—as shown by an impaired visually enhanced VOR—then spinocerebellar and Friedreich’s ataxia [141] and CANVAS [142] need to be considered.

*vHIT: potential practical pitfalls* Although vHIT can be quick and easy to do, it requires training, practice, and attention to detail [143, 144]. For example, it is important to interact with the patient throughout testing, continually exhorting her to pay attention to the fixation target (as in visual field testing), not to blink, and not to resist or try to help with the passive head turning. It is important to give head impulse stimuli over the entire magnitude range up to 300°/s peak head velocity. Testing the vertical canals requires special attention to eccentric horizontal eye position [145]. The reason it is possible to test the 3-dimensional vestibular sensory system with a 2-dimensional method, the vHIT, is that when eyes deviate horizontally so that they align with vertical impulses being delivered directly in a vertical SCC plane, the VOR is entirely vertical; torsional eye movements, which cannot be detected by the video method, are eliminated [146]. vHIT testing using a head-fixed rather than space-fixed visual target—the SHIMP paradigm [147]—gives clearer results in patients with many covert saccades and in those with only low-level residual SCC function.

*vHIT and caloric testing* Caloric testing was the mainstay of vestibular testing for a century; it still has a place in some cases with a normal vHIT. It is now proposed that vHIT should be the first test done in a patient with a suspected vestibular problem [148–150]. If the vHIT is abnormal then there is no point in asking for calorics—they will not give any more diagnostic information [148]. On the other hand, if the vHIT data are clean and truly normal, over the entire stimulus magnitude range, then it might be worth asking for calorics. For example, it seems that in MD the caloric is impaired but the vHIT is not [48, 96, 124]. One explanation for this discrepancy is that since MD preferentially causes loss of type II vestibular hair cells [151], it will preferentially impair tonic (responsible to caloric responses) rather than phasic canal signals (responsible for impulsive responses). An alternative explanation is that the caloric impairment is a hydrodynamic effect from the swelling of the endolymphatic compartment abolishing the possibility of thermal convection—the main proposed mechanism of caloric stimulation [48]. Also, in patients with recovered vestibular neuritis, the recovery might be less obvious on caloric testing than on vHIT. This means that a patient seen some time after an acute vestibular syndrome who now has a normal vHIT should have a caloric test—as it might still show a canal paresis [106, 108], indicating that it really was vestibular neuritis and there is no need for MRI.

*vHIT and VEMPs* VEMPs can give a semi-quantitative measurement of the function of each of the four otoliths—two utricles and two saccules. VEMPs combined with vHIT make it possible to test each of the 10 vestibular organs individually [152]. VEMPs are about as difficult to do as any other evoked potential test in clinical neurophysiology. There are, however, some important specific technical details to follow to record meaningful results. (a) Correct calibration of the air-conducted sound stimulus—it needs to be loud enough to be effective, and not so loud as to be unsafe [153]. (b) The stimulator for bone-conducted VEMPs can be as simple as a triggered tendon hammer; for more accuracy, an electro-mechanical vibrator such as the B&K minishaker is useful [152]. (c) Measuring background rectified EMG activation from sternomastoid muscle with cervical VEMPs makes measurement of the left/right VEMP asymmetry ratio more accurate [154]. What are some clinical situations in which VEMP testing might be useful? Consider the patient who has recovered from an acute vestibular syndrome with no impairment of vHIT, but with a canal paresis on a caloric test. Here, an absent ipsilateral ocular VEMP in the contralateral eye confirms that the patient had superior vestibular neuritis [102–104, 106, 109]. Similarly, if the patient has only an impaired posterior canal vHIT, then an absent ipsilateral cervical VEMP supports the diagnosis of inferior vestibular neuritis [101, 106]. VEMPs can also help decide if a radiologically suspected superior canal dehiscence is likely to be symptomatic [155]: if the air-conducted oVEMP from that side has a low threshold and a large amplitude, then it probably is [156–158].
